# Clinical and Nutritional Impact of a Semi-Elemental Hydrolyzed Whey Protein Diet in Patients with Active Crohn’s Disease: A Prospective Observational Study

**DOI:** 10.3390/nu13103623

**Published:** 2021-10-16

**Authors:** Blanca Ferreiro, Silvia Llopis-Salinero, Beatriz Lardies, Carla Granados-Colomina, Raimon Milà-Villarroel

**Affiliations:** 1 Digestive System Department, Hospital Universitario Virgen de la Victoria, Campus de Teatinos, S/N, 29010 Málaga, Spain; 2 Endocrinology and Nutrition Department, Hospital Universitario Infanta Leonor, 28031 Madrid, Spain; aivlis_75@hotmail.com; 3 Endocrinology and Nutrition Department, Hospital Obispo Polanco, 44002 Teruel, Spain; bealardies@gmail.com; 4 Trialance SCCL, 8051 Barcelona, Spain; cgranados@trialance.com; 5 Group Research on Wellbeing (GRoW), Blanquerna School of Health Sciences—Universitat Ramon Llull, 08022 Barcelona, Spain; raimonmv@blanquerna.url.edu

**Keywords:** semi-elemental diet, hydrolyzed whey protein, Crohn’s disease, disease activity, nutritional status, stool frequency

## Abstract

Background: Malnourishment is a common complication in patients with Crohn’s disease. Methods: An observational, prospective study was conducted to assess the nutritional status, disease activity, and stool frequency at baseline and after 12 weeks of treatment with a semi-elemental diet in patients with active Crohn’s disease. Results: A total of 144 patients with Crohn’s disease were included. The nutritional status improved after treatment, resulting in 76.1% of patients at low risk of malnourishment, 20.4% moderately malnourished, and 8.5% severely malnourished after 12 weeks of treatment. Nutritional status improvement was associated with the number of nutritional supplements. Mean albumin levels and body mass index (BMI) improved after 12 weeks of nutritional treatment (from 3.0 g/dL to 3.7 g/dL and from 20.2 kg/m^2^ to 21.1 kg/m^2^, respectively). A significant decrease in HBI was found after 12 weeks of nutritional treatment (from 10.2 to 3.7). The mean number of stools per day decreased with the 12 week semi-elemental diet (from 4.6 stools/day to 1.7 stools/day). Conclusion: In this observational study, the semi-elemental diet seemed effective in improving the nutritional status, disease activity, and stool frequency in patients with active Crohn’s disease.

## 1. Introduction

Crohn’s disease, a chronic inflammatory bowel disease (IBD) of the gastrointestinal tract, is characterized by periods of remission and recurrent relapses [1]. The most common symptoms are abdominal pain and diarrhea, whereas fatigue, weight loss, fever, anemia, or perianal lesions are less frequently recorded [1]. The incidence of Crohn’s disease is increasing worldwide, with a wide geographical variation, ranging from 0.6 to 322 cases per 100,000 persons [2]. Diverse genetic, immunological, and environmental factors contribute to the multifactorial etiology of Crohn’s disease [3]. Diet, a modifiable factor, is gaining relevance in the pathogenesis of Crohn’s disease because it modulates the intestinal microbiome, mucosal integrity, and immune system [4].

Crohn’s disease is frequently characterized by reduced oral intake, nutrient malabsorption, and a hypermetabolic state, which substantially compromise the nutritional status of patients [5]. Since malnourishment is prevalent in different clinical scenarios of Crohn’s disease—remission, active disease, and the peri-operative period [6]—preventing and correcting malnourishment is one of the current treatment goals [7]. Nutritional support can be key to prevent or correct malnourishment and osteoporosis, improve disease activity, and promote adequate development in children [6]. In different studies, enteral nutrition improved the nutritional status and inflammatory markers [8] and reduced surgical complications in patients with Crohn’s disease [9]. Accordingly, the ESPEN guideline recommends enteral nutrition as first-line treatment in the pediatric population and oral nutritional supplements during the peri-operative period when energy needs are unmet [5].

There are three types of enteral nutrition formulas depending on the nitrogen source: elemental (amino acid), semi-elemental or oligomeric (oligopeptide), and polymeric (whole protein) [6]. A systematic review concluded that corticosteroids may be more effective at inducing clinical remission than enteral nutrition in adult patients with active Crohn’s disease, but results showed very-low-quality evidence [10]. Among the studies included, four compared elemental and semi-elemental diets [11,12,13,14] and seven compared elemental vs. polymeric diets [15,16,17,18], with no differences in remission rates by the type of formula [10]. Similarly, in another systematic review, elemental and polymeric diets were equally effective at maintaining remission in quiescent patients with Crohn’s disease, while no study using a semi-elemental diet was included [7]. Conversely, enteral nutrition was considered equal or more effective than corticosteroids in pediatric patients with Crohn’s disease [10,19].

Semi-elemental enteral nutrition can be an attractive option for patients with severe disease and malnourishment because the presence of peptides and medium-chain triglycerides increases digestibility, protects mucosal integrity, and facilitates nutrient absorption [20]. Moreover, the higher palatability of semi-elemental compared with elemental diets likely improves adherence to nutritional treatment [21]. However, few recent studies have assessed the impact of semi-elemental formulas in patients with active Crohn’s disease. Therefore, we investigated the effect of a semi-elemental diet on the nutritional status, body mass index (BMI), albumin levels, disease activity, and stool frequency in patients with active Crohn’s disease.

## 2. Materials and Methods

### 2.1. Study Design

This observational, prospective multicenter study was conducted at 12 Spanish centers. The study protocol was approved by the Ethics Committee of Investigación Provincial de Málaga (Málaga, Spain) and performed in accordance with the tenets of the Declaration of Helsinki. All participants provided written informed consent before study initiation.

The effectiveness of a semi-elemental diet in patients with active Crohn’s disease was assessed after a 12 week treatment period. At Visit 1 (week 0), demographic, concomitant medications, and clinical data were collected, and nutritional status, disease activity, and the number of stools were assessed. At Visit 2 (week 12), disease activity, nutritional status, tolerance to treatment, number of stools, and treatment adherence were evaluated.

Nutritional support consisted of a semi-elemental formula with a calorie density of 1 kcal/mL, composed of hydrolyzed whey protein (HWP, 18.6%), carbohydrates (56.4%), and fats (25%; 51% as medium-chain triglycerides), without fiber. Nutritional information is provided in Supplementary Table S1. Patients at low risk of malnourishment received one bottle per day of the semi-elemental HWP diet, moderately malnourished patients received two bottles per day, and severely malnourished patients received three bottles per day.

### 2.2. Study Population

Eligible subjects were outpatients with active Crohn’s disease (Harvey–Bradshaw index, HBI, >5) and malnourishment. Exclusion criteria comprised (1) the presence of heart disease, kidney disease, or any comorbidity that could cause malnourishment, (2) diarrhea associated with antibiotics, H2-receptor antagonists or prokinetics, laxatives, or osmotically active agents, (3) *Clostridium difficile* infection, or (4) treatment with other nutritional support.

### 2.3. Study Outcomes

The objective of the study was to assess the effectiveness of the semi-elemental HWP formula at improving the nutritional status, disease activity, and stool frequency in patients with active Crohn’s disease. Other secondary objectives were to identify determinants of improvement in nutritional status, BMI, albumin, disease activity, and stool frequency and to evaluate treatment tolerance and adherence. The nutritional status of patients was evaluated with a simplified version of the Subjective Global Assessment (SGA) that considers weight, BMI, albumin levels, and dietary intake change. On the basis of this assessment, patients were categorized as at low risk of malnourishment, moderately malnourished, or severely malnourished at baseline and after 12 weeks of treatment with the semi-elemental HWP diet. Other endpoints were the change in disease activity, BMI, albumin levels, and stool frequency from baseline to week 12, potential predictors of improvement in these variables, and treatment adherence and tolerance at week 12.

Disease activity was evaluated with the HBI [22], which comprises five domains: general wellbeing, abdominal pain, number of solid stools per day, abdominal mass, and complications. General wellbeing was rated on a five-point scale (0 = very well; 4 = terrible), abdominal pain was rated on a four-point scale (0 = none; 3 = severe), and abdominal mass was rated on a four-point scale (0 = none; 3 = definite and tender). The presence of complications (arthralgia, uveitis, erythema nodosum, aphthous ulcers, pyoderma gangrenosum, anal fissure, new fistula, and abscess) received one point per item.

Bivariate analyses were used to identify significant predictors of improvement in nutritional status, BMI, albumin levels, disease activity, and stool frequency. The following factors were assessed as potential determinants of nutritional status improvement: time from diagnosis (recent/previous), previous surgery, number of supplements prescribed (one, two, or three bottles/day), and weight change. The potential associations between the improvement in BMI, albumin levels, or stool frequency and nutritional status, number of supplements prescribed, disease symptomatology (good, moderate, or severe), disease severity (remission, mild, or moderate), and adherence to prescribed semi-elemental HWP treatment were evaluated. Nutritional status, the number of supplements received per day, treatment tolerance, disease severity, and time from diagnosis were assessed as potential determinants of HBI improvement. Disease severity was classified on the basis of the HBI score as at remission (HBI < 5), mild (HBI, 5–7), or moderate (HBI, 8–16).

Adherence to the nutritional treatment was classified by the average volume consumed throughout the study over the total prescribed: all the content (200 mL/bottle), 2/3 of the content (150 mL/bottle), 1/2 of the content (100 mL/bottle), and 1/4 of the content (50 mL/bottle). Patients reported the average volume consumed throughout the study at week 12.

Tolerance to nutritional treatment was assessed by recording the frequency of nausea, vomiting, reflux, abdominal pain, flatulence, satiety, constipation, and stomach heaviness 2 h after the nutritional support intake. Patients rated the frequency of each event as ‘never’, ‘rarely’, ‘sometimes’, ‘often’, and ‘always’ at week 12. For bivariate analyses, tolerance was classified as good when no gastrointestinal symptomatology was reported or as poor when mild, moderate, or severe gastrointestinal symptomatology was registered.

### 2.4. Statistical Analyses

The IBM SPSS Statistics v.24.0 (IBM Corp, Armonk, NY, USA) was used for statistical analyses. The level of statistical significance was set at *p* < 0.05. Data distribution was tested with the Kolmogorov–Smirnov test. Categorical variables were expressed as counts and percentages, and continuous variables were expressed as means and standard deviations. Differences from baseline to week 12 were calculated using the McNemar test for categorical variables and the paired *t*-test for continuous variables. Bivariate analyses were assessed with the ANOVA test or chi-square test, as appropriate.

### 2.5. Sample Size Calculation

A sample size of 137 participants was required assuming a 5% type I (α) error, 80% power, and 20% dropout rate, and considering a nutritional status improvement in 20% of patients (from 65% of patients with malnourishment at baseline to 45% at week 12) [23,24,25,26,27].

## 3. Results

### 3.1. Study Population

A total of 144 patients were included, and 136 completed the study. The mean age in the overall population was 50 years, and 77 were men (53.5%). Among the total number of patients, 36.1% were recently diagnosed with Crohn’s disease, and 33.6% had previous surgery (Table 1).

The following concomitant treatments were reported at baseline: corticosteroids (70.1%), immunosuppressants (29.9%), biological therapy (31.9%), aminosalicylates (32.6%), antibiotics (21.5%), and other treatments (9.7%) (Table 1).

### 3.2. Nutritional Status

**Figure 1:**
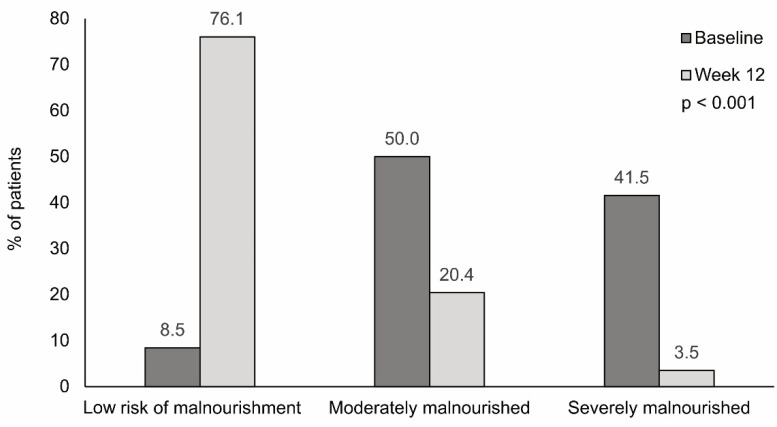
Change in nutritional status after 12 weeks of treatment with a semi-elemental hydrolyzed whey protein diet. The bar graph shows the proportion of patients at low risk of malnourishment and with moderate or severe malnourishment at baseline and after 12 weeks. The change in the nutritional status was statistically significant (*p* < 0.001).

At baseline, most patients were moderately malnourished (50%), 41.5% were severely malnourished, and 8.5% were at low risk of malnourishment. After 12 weeks of nutritional treatment, 76.1% of patients were at low risk of malnourishment, 20.4% were moderately malnourished, and only 3.5% were severely malnourished (Figure 1). Differences in the nutritional status from baseline to week 12 were significant (*p* < 0.001). The nutritional status improved in 119 (83.8%) patients, with most patients (54.6%) improving from moderate to low risk, 26.1% improving from severe to low risk, and 19.3% improving from severe to moderate malnourishment. Among those 23 patients who maintained their nutritional status, 52.2% remained at low risk of malnourishment, 26.1% remained at moderate malnourishment, and 21.7% remained at severe malnourishment. The change in the nutritional status was not associated with previous surgery or time from diagnosis (recent/previous) but depended on the number of supplements prescribed (Supplementary Table S2).

**Figure 2:**
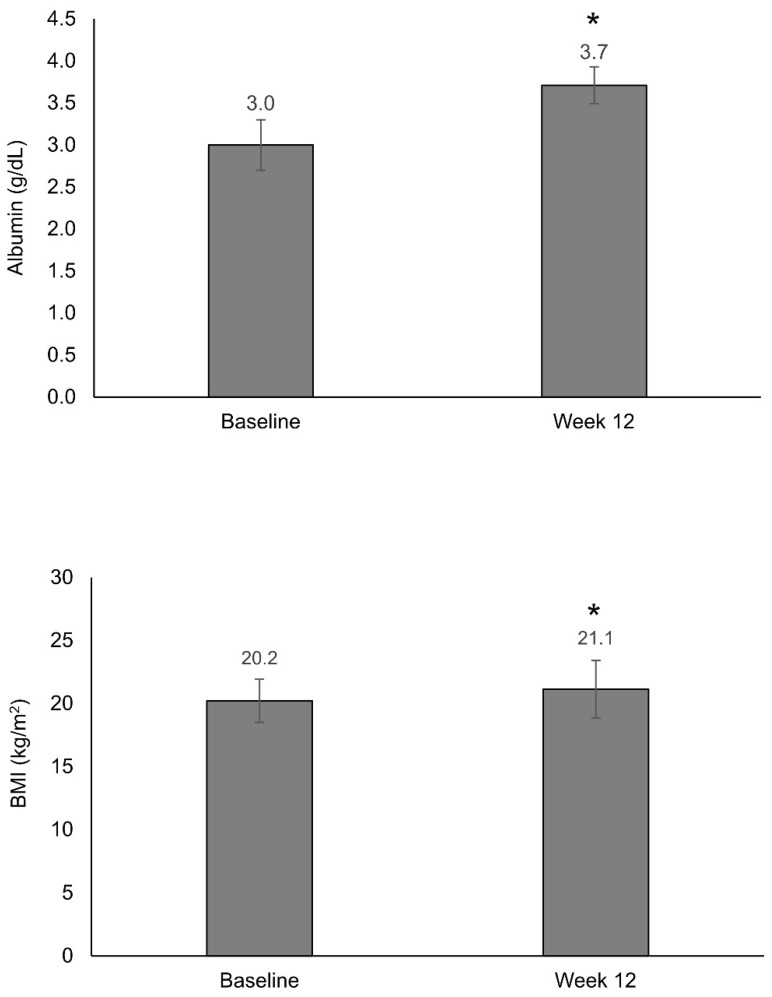
Change in body mass index and albumin levels after 12 weeks of treatment with a semi-elemental hydrolyzed whey protein diet. Bar graphs show mean (SD) albumin levels and body mass index (BMI) at baseline and after 12 weeks of treatment; * *p* < 0.001.

Mean albumin levels increased from 3.0 g/dL at baseline to 3.7 g/dL at week 12 (*p* < 0.001) (Figure 2). The increase in serum albumin levels was associated with the number of supplements received, with the highest increase being in patients treated with three supplements/day (0.89 g/dL; *p* < 0.001). Treatment adherence was a significant determinant of increased albumin levels, with an increase of 0.73 g/dL in those patients consuming the entire content vs. 0.14 g/dL in those consuming 1/2 of the content (*p* = 0.04). Disease severity (remission, mild, or moderate) and symptomatology (good, moderate, or severe) were not significant determinants of improvement in albumin levels (Supplementary Table S3).

A significant BMI increase from baseline (20.2 kg/m^2^) to week 12 (21.1 kg/m^2^) was observed (*p* = 0.002) (Figure 2). BMI change was associated with the nutritional status of patients (*p* < 0.001), being higher in those at low risk of malnourishment (increase, 1.1 kg/m^2^). BMI change was not associated with the number of supplements received per day, treatment adherence, or disease symptomatology. A significant association (*p* = 0.005) was found between BMI change and disease severity; patients with mild disease showed a higher increase vs. those with moderate disease (1.5 kg/m^2^ vs. 0.47 kg/m^2^) (Supplementary Table S4).

### 3.3. Disease Activity

**Figure 3:**
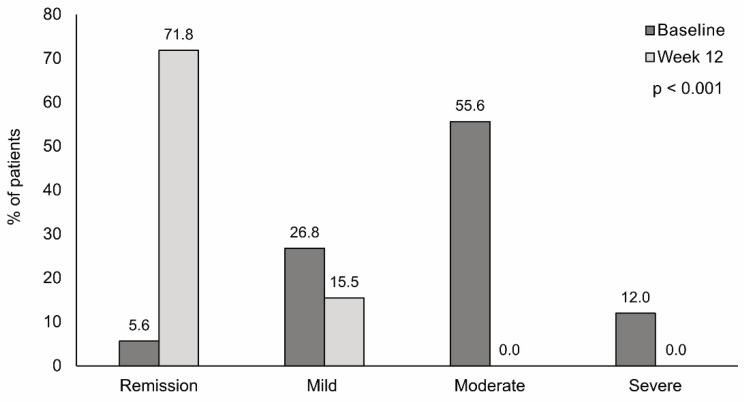
Change in Harvey–Bradshaw index (HBI) after 12 weeks of treatment with a semi-elemental hydrolyzed whey protein diet. Bar graphs show the proportion of patients in remission, and with mild, moderate, or severe disease activity at baseline and after 12 weeks of treatment. The change in HBI was statistically significant (*p* < 0.001).

Mean HBI score significantly decreased from 10.2 at baseline to 3.7 after 12 weeks (*p* < 0.001). Differences in all the HBI domains reached statistical significance, except for the incidence of pyoderma gangrenosum (*p* = 0.25) (Table 2). The proportion of patients in remission increased from 5.6% at baseline to 71.8% after 12 weeks of nutritional support (Figure 3). A significant association was found between HBI decrease and nutritional status, with a greater improvement in patients with severe malnourishment. The decrease in HBI was significantly higher in patients receiving three supplements per day (*p* < 0.001) and in those with good nutritional treatment tolerance (*p* = 0.015). HBI decrease was significantly greater in patients with a recent diagnosis than in those previously diagnosed (*p* = 0.025) (Supplementary Table S5).

### 3.4. Stool Frequency

**Figure 4:**
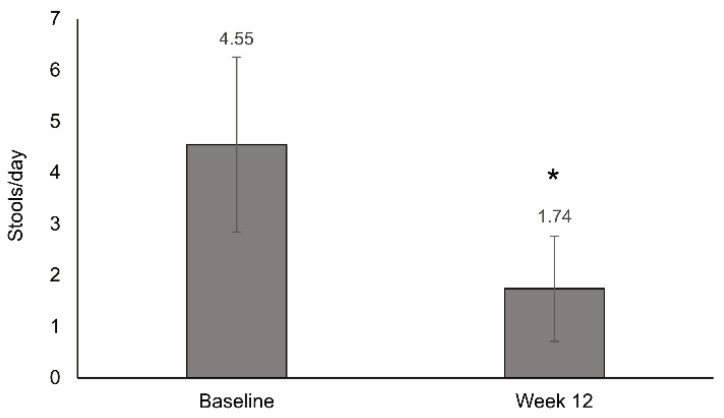
Change in stool frequency after 12 weeks of treatment with a semi-elemental hydrolyzed whey protein diet. Bar graphs show the mean (SD) number of stools per day at baseline and after 12 weeks of treatment; * *p* < 0.001.

The mean number of stools per day significantly decreased with the 12 week nutritional support (4.6 stools/day at baseline and 1.7 stools/day after 12 weeks; *p* < 0.001) (Figure 4). The change in stool frequency was not significantly associated with the nutritional status, although the decrease was higher in patients with moderate malnourishment (decrease, 3.7 stools/day) vs. those with low-risk malnourishment (decrease, 2.5 stools/day) or severe malnourishment (decrease, 2.0 stools/day). Stool frequency was independent of the number of supplements taken per day and adherence to the prescribed nutritional treatment. Disease symptomatology was significantly associated with stool frequency change; the number of stools per day decreased in patients with good (decrease, 2.8 stools/day) and moderate symptomatology (decrease, 2.8 stools/day) and increased in those with severe symptomatology (increase, 1.8 stools/day). A significant association was found between disease severity and change in stool frequency, with a decrease of 3.3, 1.8 and 1.3 stools/day for patients in remission and with mild and moderate disease, respectively (*p* < 0.012) (Supplementary Table S6).

### 3.5. Tolerance

**Figure 5:**
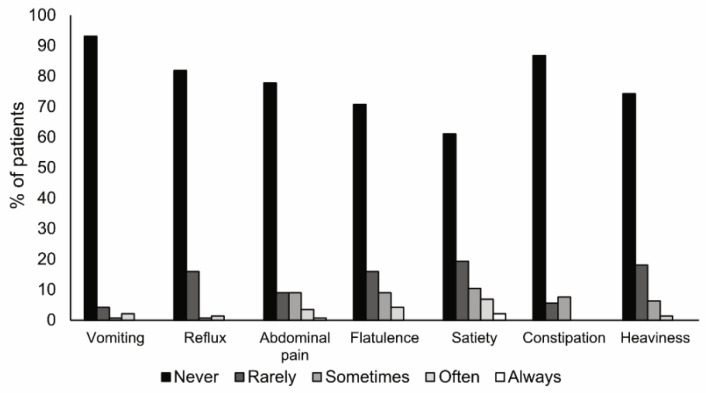
Frequency of tolerance symptoms after 12 weeks of treatment with a semi-elemental hydrolyzed whey protein diet. The bars show the frequency of nausea, vomiting, reflux, abdominal pain, flatulence, satiety, and constipation.

Nutritional support tolerance was considered good by 77.4% of patients, whereas 19.7% reported moderate gastrointestinal symptomatology after receiving the semi-elemental HWP diet. Most symptoms were rated as ‘never’ or ‘rarely’ present by the patients: 97.3% for vomiting, 97.9% for reflux, 86.8% for abdominal pain, 86.8% for flatulence, 80.5% for satiety, 92.4% for constipation, and 92.4% for heaviness (Figure 5).

Regarding treatment adherence, 80.7% of patients reported that they consumed the total content prescribed, 14.3% consumed 2/3 of the content prescribed, 3.6% consumed 1/2 of the content prescribed, and 1.4% consumed 1/4 of the content prescribed.

## 4. Discussion

In this observational study, the semi-elemental HWP diet was associated with improved nutritional status, improved disease activity, and reduced frequency of stools. Although the efficacy of nutritional support in patients with Crohn’s disease has been largely shown [7,10], these formulations are currently underused in many countries. Despite the significant efforts made to improve the palatability of formulations, unpalatability is still one of the main perceived barriers by patients and physicians [28].

The rates of moderate/severe malnourishment at baseline (91.5%) found in our study are in agreement with the high prevalence of malnourishment reported in patients with active and quiescent Crohn’s disease [6], and reinforce the importance highlighted by the ESPEN guideline of screening and correcting malnourishment in patients with IBD [5]. After 12 weeks of nutritional treatment, 83.8% of patients improved their nutritional status (even from severe to low risk of malnourishment), resulting in only 23.9% of patients being moderately/severely malnourished at the end of follow-up. Given the wide interpatient variability, it would be interesting to identify the clinical profile of patients achieving greater improvement and of those remaining at moderate or severe malnourishment after 12 weeks of nutritional treatment. In this context, bivariate analyses showed that the number of supplements taken and adherence to the semi-elemental HWP diet were significantly associated with improved nutritional status. Although we cannot establish a direct cause–consequence relationship, the association with adherence or number of supplements points out the key role of the semi-elemental HWP diet in improving the nutritional status, as previously observed in Crohn’s disease, pancreatitis, human immunodeficiency virus (HIV) [21], and cancer patients treated with the same semi-elemental HWP formula [29]. In this study, patients were categorized by nutritional status (low risk, moderately, or severely malnourished) to assess the level of change, as opposed to previous studies using only albumin levels and body weight as indicators of nutritional status [11,12,13].

The improvement in nutritional status after 12 weeks of nutritional treatment is in line with the significant increase in mean albumin levels (from 3.0 g/dL to 3.7 g/dL) and BMI (from 20.2 kg/m^2^ to 21.1 kg/m^2^). The number of supplements received and adherence with the prescribed nutritional treatment significantly correlated with albumin increase, whereas nutritional status and disease severity were significantly associated with BMI increase. These differential correlations could imply that the change in albumin levels is more closely related to the nutritional status of patients, while variations in BMI are more dependent on disease characteristics. However, this assumption requires further confirmation. In this context, BMI, fat mass, and lean body mass have been previously correlated with disease activity and duration in IBD [30]. The level of change in albumin and weight in our study is in keeping with that observed in other studies (an increase of 0.3 g/dL and of 1 kg, respectively) [11,12], although we cannot directly compare these results because of the different design and formulas used.

One of the most important findings of the study is the significant improvement in disease activity observed after 12 weeks of nutritional treatment. The mean HBI score corresponded to moderate disease activity at baseline (score of 10.1) and to disease remission (score of 3.7) after 12 weeks of nutritional support [31]. In analyzing this result, we should consider that concomitant treatments received during the study could have influenced HBI improvement. However, the improvement in disease activity is in agreement with previous studies showing the effectiveness of semi-elemental formulas in inducing clinical remission [10,11,12,13] and of enteral nutrition diets in maintaining clinical remission in patients with quiescent Crohn’s disease [7]. The efficacy of semi-elemental formulas in inducing clinical remission found in previous studies was comparable to that of elemental diets [11,13] and of corticosteroids [32], but semi-elemental diets were better tolerated [11] and presented fewer side effects [32]. Again, although causality cannot be inferred, the fact that HBI improvement was associated with nutritional status, the number of supplements, and nutritional treatment tolerance points to the potential role of this semi-elemental HWP diet in improving disease activity. One of the possible causes of the promising results on nutritional status and disease activity found in our study is the increased digestibility of HWP as a source of proteins. Moreover, HWPs are important sources of bioactive peptides, which are involved in a wide range of biological processes, including intestinal anti-inflammatory activities [33].

A significant reduction in the number of stools per day was also observed after 12 weeks of nutritional treatment (from 4.6 stools/day to 1.7 stools/day). Stool frequency was not dependent on the nutritional status, the number of supplements taken per day, or adherence to the prescribed treatment, but was associated with disease symptomatology and severity. These results could indicate that the reduction in stool frequency could be associated with the improved disease activity observed. The lack of fiber content in the diet composition and the high digestibility of the semi-elemental HWP diet could have also contributed to the improvement in stool frequency. The importance of stool frequency improvement is highlighted by the fact that diarrhea is a common cause of malnourishment in IBD [30], as well as because it is one of the factors considered to classify disease severity by the HBI or the CDAI [31]. Despite the importance of stool frequency in Crohn’s disease, this study is one of the few including this variable in the analysis [34,35].

Tolerance to the semi-elemental HWP diet was rated as good by 79.4% of patients, with satiety, flatulence, or abdominal pain being the symptoms more frequently reported, although still at a low frequency. In line with these results, 80.7% of patients reported consuming the total nutritional content prescribed. Of note, high tolerance and adherence rates were previously observed in cancer patients treated with the same semi-elemental HWP formula [29]. Tolerance and adherence are two key outcomes in studies with nutritional supplements, as the most common barriers identified in trials with enteral nutrition are the high dropout rates due to poor tolerance and unpalatable formulations [6,10]. Thus, the increased digestibility of a semi-elemental HWP formula and the higher palatability compared with conventional elemental formulations could have contributed to these good adherence and tolerance results [36]. However, we should analyze these results cautiously given the lack of a comparator and considering that patients subjectively reported these variables.

The main limitation of the study was the lack of comparator, which did not allow us to know the actual effect of the semi-elemental HWP diet in our patient cohort. Although the results observed are in line with those previously reported with semi-elemental diets, we cannot rule out that other variables such as concomitant treatments were, to some extent, responsible for the outcomes observed. Another limitation is that, unlike most previous studies, we used the HBI to measure disease activity, whereas validation with the CDAI would have been desirable. The HBI is a simple index to rate Crohn’s disease severity that does not require a prospective 7 day collection of data, and that has a 93% correlation with the CDAI [37].

Despite these limitations, this study, comprising 136 patients, is one of the largest investigating the effect of nutritional support in patients with active Crohn’s disease [11,12,13,18,23,38]. Another strength of the study is the comprehensive assessment of patients, including nutritional status, albumin, weight, disease activity, stool frequency, adherence, and tolerance.

## 5. Conclusions

The semi-element HWP diet seemed effective in improving the nutritional status, disease activity, and frequency of stools in patients with active Crohn’s disease.

## Figures and Tables

**Table 1:** Clinical and demographic characteristics of study participants.

	*N* = 144
Age (years), mean (SD)	50 (18)
Sex, *n* (%)	
Men	77 (53.5)
Women	67 (46.5)
Weight (kg), mean (SD)	56.8 (12.3)
BMI (kg/m^2^), mean (SD)	20.2 (3.4)
Crohn’s disease diagnosis, *n* (%)	
Recent	52 (36.1)
Previous	92 (63.9)
Previous surgery, *n* (%) (*n* = 143)	48 (33.6)
Treatment ^a^, *n* (%)	
Corticosteroids	101 (70.1)
Immunosuppressants	43 (29.9)
Biological therapy	46 (31.9)
Aminosalicylates	47 (32.6)
Antibiotics	31 (21.5)
Other treatments	14 (10)

Data are expressed as the mean (SD) or *n* (%). ^a^ Patients could have received more than one treatment. BMI, body mass index; SD, standard deviation.

**Table 2:** Change in Harvey–Bradshaw index (HBI) score from baseline to week 12.

Variable		Change	*p*-Value
General wellbeing	Very well	32.60%	<0.001
	Slightly below	29.20%	
	Poor	−39.60%	
	Very poor	−18.70%	
	Terrible	−3.50%	
Abdominal pain	None	41.70%	<0.001
	Mild	8.30%	
	Moderate	−36.10%	
	Severe	−13.90%	
Abdominal mass	None	33.30%	<0.001
	Dubious	−14.90%	
	Definite	−8.50%	
	Definite and tender	−9.90%	
Number of stools		Mean difference (−2.8)	<0.001
Complications	Arthralgia	−11.10%	0.004
	Uveitis	−9.00%	<0.001
	Erythema nodosum	−4.80%	<0.001
	Aphthous ulcers	−7.60%	<0.001
	Pyoderma gangrenosum	−2.10%	0.25
	Anal fissure	−13.90%	<0.001
	New fistula	−15.20%	<0.001
	Abscess	−8.30%	<0.001

## Data Availability

The data presented in this study are available on request from the corresponding author.

## Supplementary Materials

The following are available online at https://www.mdpi.com/article/10.3390/nu13103623/s1: Supplementary Table S1. Nutritional information of the semi-elemental hydrolyzed whey protein diet without fiber; Supplementary Table S2. Bivariate analysis of potential factors associated with nutritional status improvement; Supplementary Table S3. Bivariate analysis of potential factors associated with albumin level improvement; Supplementary Table S4. Bivariate analysis of potential factors associated with body mass index improvement; Supplementary Table S5. Bivariate analysis of potential factors associated with HBI improvement; Supplementary Table S6. Bivariate analysis of potential factors associated with stool frequency improvement.
