# Supplementary material for: Clinical and Nutritional Impact of a Semi-Elemental Hydrolyzed Whey Protein Diet in Patients with Active Crohn’s Disease: A Prospective Observational Study
Source: Nutrients. 2021 Oct 16;13(10):3623. doi: 10.3390/nu13103623 (PMC8538212; doi:10.3390/nu13103623)
Supplement: Supplementary file 1 [file nutrients-13-03623-s001.zip › nutrients-1413359-supplementary.pdf]

## SUPPLEMENTARY TABLES

**Supplementary Table S1.** Nutritional information of the semi-elemental whey-hydrolyzed protein diet without fiber.

| Average content | | Bottle (200 ml) |
|---|---|---|
| **Energy value** | kJ (kcal) | 840 (200) |
| **Fat** | g | 5.6 |
| Saturated fatty acids | g | 2.9 |
| Medium chain triglycerides | g | 2.7 |
| Monounsaturated fatty acids | g | 1.6 |
| Polyunsaturated fatty acids | g | 1.1 |
| **Carbohydrate** | g | 28.2 |
| Sugars | g | 10.0 |
| Lactose | g | ≤0.2 |
| **Protein** | g | 9.3 |
| **Fibre** | g | <0.2 |

**Supplementary Table S2.** Bivariate analysis of potential factors associated with nutritional status improvement.

| | | | | p |
|---|---|---|---|---|
| **Diagnosis** | **Recent** | | **Previous** | 0.447 |
| Maintenance low-risk malnourishment | 5 (9.8%) | | 7 (7.7%) | |
| Maintenance moderate malnourishment | 2 (3.9%) | | 4 (4.4%) | |
| Maintenance severe malnourishment | 2 (3.9%) | | 3 (3.3%) | |
| 1-level improvement (severe to moderate) | 5 (9.8%) | | 18 (19.8%) | |
| 2-level improvement (severe to low-risk malnourishment) | 16 (31.4%) | | 15 (16.5%) | |
| 1-level improvement (moderate to low-risk malnourishment) | 21 (41.2%) | | 44 (48.4%) | |
| **Surgery** | **Yes** | | **No** | 0.998 |
| Maintenance low-risk malnourishment | 2 (4.3%) | | 10 (10.6%) | |
| Maintenance moderate malnourishment | 0 (0.0%) | | 6 (6.4%) | |
| Maintenance severe malnourishment | 2 (4.3%) | | 3 (3.2%) | |
| 1-level improvement (severe to moderate) | 11 (23.4%) | | 11 (11.7%) | |
| 2-level improvement (severe to low-risk malnourishment) | 11 (23.4%) | | 20 (21.3%) | |
| 1-level improvement (moderate to low-risk malnourishment) | 21 (44.7%) | | 44 (46.8%) | |
| **Number of supplements** | **1 bottle/day** | **2 bottle/day** | **3 bottle/day** | <0.001 |
| Maintenance low-risk malnourishment | 5 (100%) | 7 (9.6%) | 0 (0.0%) | |
| Maintenance moderate malnourishment | 0 (0.0%) | 5 (6.8%) | 1 (1.6%) | |
| Maintenance severe malnourishment | 0 (0.0%) | 0 (0.0%) | 5 (7.8%) | |
| 1-level improvement (severe to moderate) | 0 (0.0%) | 0 (0.0%) | 23 (35.9%) | |
| 2-level improvement (severe to low-risk malnourishment) | 0 (0.0%) | 0 (0.0%) | 31 (48.4%) | |
| 1-level improvement (moderate to low-risk malnourishment) | 0 (0.0%) | 61 (83.6%) | 4 (6.3%) | |
| **Weight** | **Loss** | | **Gain** | <0.001 |
| Maintenance low-risk malnourishment | 5 (25.0%) | | 7 (5.9%) | |
| Maintenance moderate malnourishment | 3 (15.0%) | | 3 (2.5%) | |

| | | |
|---|---|---|
| Maintenance severe malnourishment | 5 (25.0%) | 0 (0.0%) |
| 1-level improvement (severe to moderate) | 3 (15.0%) | 20 (16.8%) |
| 2-level improvement (severe to low-risk malnourishment) | 1 (5.0%) | 30 (25.2%) |
| 1-level improvement (moderate to low-risk malnourishment) | 3 (15.0%) | 59 (49.6%) |

P-values were calculated with the Chi-square test.

**Supplementary Table S3.** Bivariate analysis of potential factors associated with albumin levels improvement.

| | | | | | p |
|---|---|---|---|---|---|
| **Nutritional status** | **Low-risk malnourishment** | **Moderate malnourishment** | **Severe malnourishment** | | <0.001 |
| Change in albumin levels | 0.52 | 0.50 | 0.93 | | |
| **Number of supplements** | **1 bottle/day** | **2 bottle/day** | **3 bottle/day** | | <0.001 |
| Change in albumin levels | 0.06 | 0.54 | 0.89 | | |
| **Gastrointestinal symptomatology** | **Good** | **Moderate** | **Severe** | | 0.073 |
| Change in albumin levels | 0.74 | 0.49 | 0.5 | | |
| **Disease severity** | **Remission** | **Mild** | **Moderate** | | 0.877 |
| Change in albumin levels | 0.69 | 0.71 | 0.63 | | |
| **Adherence** | **Whole content** | **2/3 content** | **1/2 content** | **1/4 content** | 0.032 |
| Change in albumin levels | 0.73 | 0.6 | 0.14 | 0.2 | |

P-values were calculated with the ANOVA test.

**Supplementary Table S4.** Bivariate analysis of potential factors associated with body mass index improvement.

| | | | | | p |
|---|---|---|---|---|---|
| **Nutritional status** | **Low-risk malnourishment** | **Moderate malnourishment** | **Severe malnourishment** | | <0.001 |
| BMI change | 1.14 | 0.99 | -0.48 | | |
| **Number of supplements** | **1 bottle/day** | **2 bottle/day** | **3 bottle/day** | | 0.275 |
| BMI change | 0.55 | 0.83 | 1.04 | | |
| **Gastrointestinal symptomatology** | **Good** | **Moderate** | **Severe** | | 0.294 |
| BMI change | 1.04 | 1.33 | 0.49 | | |
| **Disease severity** | **Remission** | **Mild** | **Moderate** | | 0.005 |
| BMI change | 0.87 | 1.51 | 0.47 | | |
| **Adherence** | **Whole content** | **2/3 content** | **1/2 content** | **1/4 content** | 0.368 |
| BMI change | 0.99 | 1.56 | 0.66 | 0.35 | |

BMI, body mass index. P-values were calculated with the ANOVA test.

**Supplementary Table S5.** Bivariate analysis of potential factors associated with HBI improvement.

| | | | | p |
|---|---|---|---|---|
| **Nutritional status** | **Low-risk malnourishment** | **Moderate malnourishment** | **Severe malnourishment** | |
| HBI change | -38.33 | -48.87 | -81.17 | <0.001 |
| **Diagnosis** | **Recent** | | **Previous** | 0.025 |
| HBI worsening | 9.6% | | 1.1% | |
| HBI improvement | 90.4% | | 98.9% | |

P-values were calculated with the ANOVA test for HBI change or the Chi-square test for diagnosis.

**Supplementary Table S6.** Bivariate analysis of potential factors associated with stool frequency improvement.

| | | | | | p |
|---|---|---|---|---|---|
| **Nutritional status** | **Low-risk malnourishment** | **Moderate malnourishment** | **Severe malnourishment** | | |
| Stool frequency change | -2.53 | -3.72 | -2 | | 0.135 |
| **Number of supplements** | **1 bottle/day** | **2 bottle/day** | **3 bottle/day** | | |
| Stool frequency change | -1.40 | -2.35 | -3.42 | | 0.072 |
| **Gastrointestinal symptomatology** | **Good** | **Moderate** | **Severe** | | 0.012 |
| Stool frequency change | -2.82 | -2.78 | 1.75 | | |
| **Disease severity** | **Remission** | **Mild** | **Moderate** | | 0.012 |
| Stool frequency change | -3.26 | -1.82 | -1.33 | | |
| **Adherence** | **Whole content** | **2/3 content** | **1/2 content** | **1/4 content** | 0.399 |
| Stool frequency change | -2.79 | -2.40 | -4.20 | 0.0 | |

P-values were calculated with the ANOVA test
